# Metastatic Retroperitoneal Leiomyosarcoma to the Right Lower Eyelid Presenting as a Chalazion

**DOI:** 10.18502/jovr.v20.16421

**Published:** 2025-09-09

**Authors:** Kevin Eid, Shwetha Mudalegundi, Melissa Yao, Alen Eid, Matthew Szarko, John Nguyen

**Affiliations:** ^1^Oakland University William Beaumont School of Medicine, Rochester, MI, USA; ^2^John A. Moran Eye Center, University of Utah, Salt Lake City, UT, USA; ^3^Wilmer Eye Institute, Johns Hopkins University, Baltimore, MD, USA; ^4^David Geffen School of Medicine at University of California at Los Angeles, Los Angeles, CA, USA; ^5^West Virginia University Eye Institute, Morgantown, WV, USA; ^6^West Virginia University Medicine Ruby Memorial Hospital, Morgantown, WV, USA

**Keywords:** Chalazion; Eyelid, Leiomyosarcoma, Metastatic, Oculoplastic Surgery

## Abstract

**Purpose:**

Leiomyosarcoma (LMS) is an aggressive tumor with a high metastatic rate that rarely metastasizes to the periocular region.

**Methods:**

A 50-year-old male with a previous two-year history of primary stage IV LMS presented with metastatic retroperitoneal LMS, which was initially incorrectly described as an eyelid chalazion refractory to medical management. An excisional biopsy sent to pathology revealed metastatic retroperitoneum LMS. There was resolution of ocular irritation following biopsy, and an oncology referral was made.

**Conclusion:**

This case of metastatic LMS to the eyelid mimicking a chalazion is rare, as only six other cases have been described previously. Our case contributes to this discussion by highlighting the importance of considering metastatic disease and performing a full-thickness biopsy in a patient presenting with a non-resolving eyelid chalazion. Recognizing tumor spread to the eyelid can be an important step in the diagnosis, surveillance, and management of metastatic LMS.

##  INTRODUCTION

Leiomyosarcoma (LMS) is a rare but highly aggressive soft tissue sarcoma of the smooth muscle.^[1]^ While the common primary sites of LMS include the uterus, stomach, intestines, retroperitoneum, and large blood vessels, the disease has been characterized as originating and metastasizing anywhere in the body because of hematogenous spread and the prevalence of smooth muscle-containing blood vessels in most tissues.^[2]^ Due to its pervasive nature, LMS frequently can mimic other kinds of neoplastic or non-neoplastic pathology, clinically based on the location of the primary site or site of metastasis. Ocular manifestations of the disease can occur, but have been mostly reported as metastasis to the conjunctiva or the orbit.^[3,4,5,6,7,8]^ Other ocular sites of involvement previously reported include the eyelid, the choroid, and the iris, but these reports are small in number.^[9,10,11,12,13,14]^ Symptoms of LMS in these ocular sites have manifested as foreign body sensation, loss of visual acuity, proptosis, swelling, and in the case of eyelid metastasis, persistent chalazion and mechanical ectropion. Eyelid neoplasms are relatively common, but metastatic lesions in the eyelid are not, and only make up < 1% of all eyelid neoplasms, usually in patients with known malignancy.^[15]^ The most common site of ocular metastases is the choroid, followed by the orbit.^[15]^ There have been reports of other ocular adnexal metastases, including those originating from breast cancer, lung cancer, melanoma, and renal cell carcinoma, which have presented similarly to previously reported LMS ocular lesions.^[15]^

In this report, we describe a case of a metastatic retroperitoneal LMS presenting as an eyelid chalazion refractory to medical management. Given the very rare incidence of such lesions without any other ocular involvement, we aim to share the imaging and histopathological characteristics of this case.

##  CASE PRESENTATION

**Figure 1:**
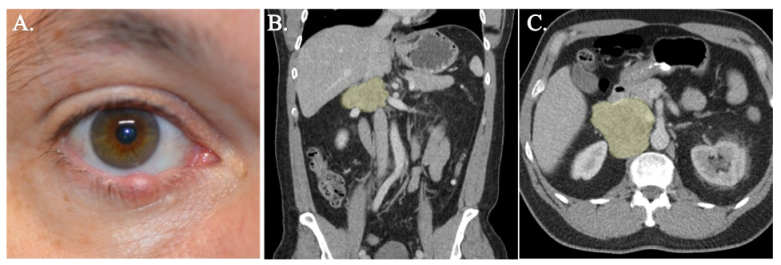
(A) External photography of the right adnexa demonstrating a firm, elevated lesion that involves the eyelid margin. (B & C) Venous phase contrast-enhanced computerized tomography of the abdomen demonstrating a large soft tissue mass within the right retroperitoneum in coronal (B) and transverse (C) views represented by a yellow overlay.

**Figure 2:**
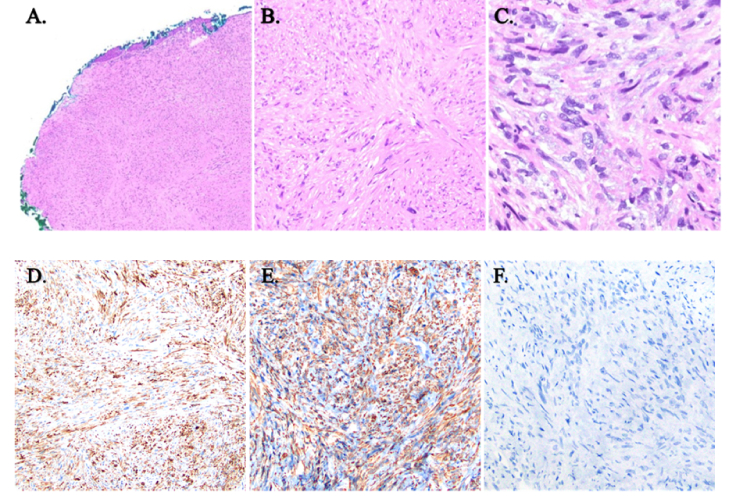
Biopsied eyelid lesion. (A) Subepithelial spindle cell proliferation extending to the margins. (B) The presence of a fascicular growth pattern is common in sarcomas. (C) There are notable pleomorphic cells, which include atypical hyperchromatic cells along with apoptotic tumor cells and nuclear debris. Immunohistochemical staining of the biopsied lesion. (D & E) Demonstration of positive desmin (D) and smooth muscle actin stain (E). (F) Negative staining of pan cytokeratin that is common to other epithelial-based tumors, e.g., squamous cell carcinoma.

A 50-year-old male with a two-year history of primary stage IV LMS of the right retroperitoneum presented with a slow-growing lump on the right lower eyelid for 12 months. Oncologic history included LMS disseminated to the right kidney, liver, and colon, for which he had undergone inferior vena cava (IVC) resection with graft reconstruction, right nephrectomy and adrenalectomy, wedge resection of the liver mass, and endoscopic resection of metastatic colon polyps 13 months earlier. Adjuvant chemotherapy was deferred due to the high risk of nephrotoxicity in this patient with a kidney transplant secondary to concomitant chronic kidney disease. The patient's primary complaint was lid irritation with a foreign body sensation, which was most noticeable when wearing his contact lenses. He was treated for a presumed chalazion with ophthalmic antibiotic-corticosteroid drops without improvement in symptoms. Ophthalmic examination was only significant for an 8 mm × 5 mm indurated and erythematous nodule on the right lower lid margin without madarosis or ulceration [Figure 1]. An excisional biopsy revealed atypical spindle cells with high mitotic activity, which stained positive for smooth muscle actin and desmin and negative for S100 and CD34, consistent with metastatic retroperitoneum LMS when compared to prior specimens [Figure 2]. He had resolution of ocular irritation following the biopsy; however, he deferred additional treatment on follow-up after consulting with the oncology service regarding the prognosis.

**Table 1:** Summary and comparison of documented leiomyosarcoma eyelid lesions

	**Present case**	**Esmaeli et al^[11]^**	**Vichare et al^[9]^**	**Wrede et al^[16]^**	**Rohrbach et al^[17]^**	**Mansour et al^[12]^**	**Carillo-Correa et al^[10]^**
Age	50	56	60	28	32	NA	24
Sex	M	F	M	F	M	M	F
Location of lid lesion	RLL, medial margin	LUL, medial margin	RLL	RLL, lateral margin	LUL	NA	RUL
Presenting ocular complain	FBS, persistent chalazion	Persistent chalazion	Mechanical ectropion, swelling, × 2 lid lesions	Painless	Painless	NA	Persistent chalazion, painless
Duration of eye complaint	12M	< 1M	4M	Rapidly growing	NA	NA	6M
Lid pre-biopsy diagnosis	Chalazion	Chalazion	Sebaceous gland carcinoma	NA	NA	NA	NA
Previously known history of LMS	Yes	Yes	No	Yes	Yes	NA	No
Primary LMS location	IVC/ Retroperitoneum	Distal esophagus	Unknown	Neck	Abdomen	Hand	Eyelid lesion
Time from LMS diagnosis to lid lesion	12M	5M	NA	21M	12M	NA	NA
Prior LMS systemic treatment	None	Chemotherapy	NA	Radiotherapy	Chemotherapy	NA	None
Treatment of lid lesion	Excisional biopsy	Drainage, then excisional biopsy	Shave biopsy	Excisional biopsy	Excisional biopsy	NA	Excisional biopsy
Outcome	Resolution of FBS, deferred additional treatment	Resolution of lesion, progression to bony metastasis	Brain metastasis, SAH, palliative chemotherapy and radiation	Brain metastasis	NA	NA	Resolution of cutaneous epidermoid LMS lesion
Prognosis from onset of lid lesion	Patient alive 3M out	3M, death	NA	9M, death	4M, death	NA	Patient alive after 2Y follow-up
${}^{*}$ In the case reported by Carillo-Correa et al, cutaneous epidermoid LMS was discovered only in the eyelid. FBS, foreign body sensation; LMS, leiomyosarcoma; LUL, left upper lid; RLL, right lower lid; RUL, right upper lid; SAH, subarachnoid hemorrhage; M, male; F, female; NA, not applicable							

##  DISCUSSION

This case of an isolated metastatic LMS to the right lower eyelid mimicking an innocuous chalazion is rare, as only six other cases have been described in the literature [Table 1]. The table highlights that the clinical presentation of these cases varies considerably in appearance, location within the eyelid, speed of growth, distortion of eyelid structures, and inflammatory nature. In 6/7 documented cases, including ours, by the time an observable lesion is present on the eyelid, disseminated disease is already present. In 3/7 documented cases, mortality is observed in < 12 months from the onset of a lid lesion. In only one of the five cases, LMS was initially diagnosed due to biopsy of an eyelid lesion.^[9]^ In the other cases, the discovery of the eyelid LMS post-biopsy was evidence of known LMS disease that had progressed. Patients with eyelid metastatic lesions often have a poor systemic prognosis, with an 88% survival at 3 months and 67% at 12 months of follow-up. Patients with multiple metastases also carry a worse prognosis, regardless of treatment.^[15]^ The treatment modalities for patients with eyelid neoplasms include excisional biopsy, external beam radiation therapy (EBRT), systemic chemotherapy/immunotherapy, and observation. The treatment selected depends on clinical features such as shape, number, location, and systemic factors. For example, if undergoing systemic chemotherapy/immunotherapy, the eyelid lesion can be observed for response, whereas EBRT is used to treat those with multiple eyelid lesions.^[15]^

In summary, our findings emphasize the importance of considering metastatic disease and full-thickness biopsy in patients presenting with a common and typically non-malignant lesion, such as a non-resolving eyelid chalazion, particularly when there is no distortion of eyelashes or discharge. Thus, recognizing tumor dissemination to the eyelid can be an important step in the diagnosis, surveillance, and management of metastatic LMS.

##  Financial Support and Sponsorship

None.

##  Conflicts of Interest

None.
